# *Ascochyta rabiei*: A threat to global chickpea production

**DOI:** 10.1111/mpp.13235

**Published:** 2022-07-01

**Authors:** Ritu Singh, Kamal Kumar, Savithri Purayannur, Weidong Chen, Praveen Kumar Verma

**Affiliations:** ^1^ Plant Immunity Laboratory National Institute of Plant Genome Research (NIPGR) New Delhi India; ^2^ Department of Plant Molecular Biology University of Delhi (South Campus) New Delhi India; ^3^ Department of Entomology and Plant Pathology North Carolina State University Raleigh North Carolina USA; ^4^ Grain Legume Genetics and Physiology Research Unit, USDA Agricultural Research Service, and Department of Plant Pathology Washington State University Pullman Washington USA; ^5^ Plant Immunity Laboratory, School of Life Sciences Jawaharlal Nehru University New Delhi India

**Keywords:** Ascomycota, fungicide resistance, host resistance, necrotrophic fungus

## Abstract

The necrotrophic fungus *Ascochyta rabiei* causes Ascochyta blight (AB) disease in chickpea. *A. rabiei* infects all aerial parts of the plant, which results in severe yield loss. At present, AB disease occurs in most chickpea‐growing countries. Globally increased incidences of *A. rabiei* infection and the emergence of new aggressive isolates directed the interest of researchers toward understanding the evolution of pathogenic determinants in this fungus. In this review, we summarize the molecular and genetic studies of the pathogen along with approaches that are helping in combating the disease. Possible areas of future research are also suggested.

**Taxonomy:**

kingdom Mycota, phylum Ascomycota, class Dothideomycetes, subclass Coelomycetes, order Pleosporales, family Didymellaceae, genus *Ascochyta*, species *rabiei.*

**Primary host:**

*A. rabiei* survives primarily on *Cicer* species.

**Disease symptoms:**

*A. rabiei* infects aboveground parts of the plant including leaves, petioles, stems, pods, and seeds. The disease symptoms first appear as watersoaked lesions on the leaves and stems, which turn brown or dark brown. Early symptoms include small circular necrotic lesions visible on the leaves and oval brown lesions on the stem. At later stages of infection, the lesions may girdle the stem and the region above the girdle falls off. The disease severity increases at the reproductive stage and rounded lesions with concentric rings, due to asexual structures called pycnidia, appear on leaves, stems, and pods. The infected pod becomes blighted and often results in shrivelled and infected seeds.

**Disease management strategies:**

Crop failures may be avoided by judicious practices of integrated disease management based on the use of resistant or tolerant cultivars and growing chickpea in areas where conditions are least favourable for AB disease development. Use of healthy seeds free of *A. rabiei*, seed treatments with fungicides, and proper destruction of diseased stubbles can also reduce the fungal inoculum load. Crop rotation with nonhost crops is critical for controlling the disease. Planting moderately resistant cultivars and prudent application of fungicides is also a way to combat AB disease. However, the scarcity of AB‐resistant accessions and the continuous evolution of the pathogen challenges the disease management process.

**Useful websites:**

https://www.ndsu.edu/pubweb/pulse‐info/resourcespdf/Ascochyta%20blight%20of%20chickpea.pdf https://saskpulse.com/files/newsletters/180531_ascochyta_in_chickpeas‐compressed.pdf http://www.pulseaus.com.au/growing‐pulses/bmp/chickpea/ascochyta‐blight http://agriculture.vic.gov.au/agriculture/pests‐diseases‐and‐weeds/plant‐diseases/grains‐pulses‐and‐cereals/ascochyta‐blight‐of‐chickpea http://www.croppro.com.au/crop_disease_manual/ch05s02.php https://www.northernpulse.com/uploads/resources/722/handout‐chickpeaascochyta‐nov13‐2011.pdf http://oar.icrisat.org/184/1/24_2010_IB_no_82_Host_Plant https://www.crop.bayer.com.au/find‐crop‐solutions/by‐pest/diseases/ascochyta‐blight

## Graphical abstract

This study presents a detailed overview of the pathology of a devastating necrotrophic fungus, *Ascochyta rabiei*, that infects all the aerial parts of chickpea and causes Ascochyta blight disease.

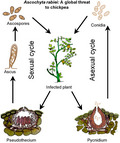

## 1 INTRODUCTION

Legumes are a vital source of protein, dietary fibre, carbohydrates, and minerals. Apart from their nutritional value, legumes also increase soil fertility through their unique ability to biologically fix atmospheric nitrogen. Ascochyta blight (AB) disease, caused by *Ascochyta* spp., is the major fungal disease constraint in several cool‐season legumes, resulting in serious yield loss globally. *Ascochyta* species, namely *Ascochyta rabiei* (causing AB disease of chickpea), *Ascochyta lentis* (causing AB disease of lentil), and *Ascochyta fabae* (causing AB disease of faba bean), cause diseases in a host‐specific manner (Kim & Chen, 2019; Taylor & Ford, 2007).

*A. rabiei* (syn. *Phoma rabiei*), also known by its teleomorph name *Didymella rabiei* (syn. *Mycosphaerella rabiei*) (Aveskamp et al., 2010; de Gruyter et al., 2009), is a haploid, heterothallic fungus (two forms at a single mating‐type locus) of phylum Ascomycota. It causes AB disease in chickpea, which is an important cool‐season legume. Being a necrotrophic fungus, *A. rabiei* kills host tissue and thrives in the dead tissue, causing serious impediments to chickpea production. Initially, the pathogen was named *Phyllosticta rabiei* (Labrousse, 1930) as Labrousse did not see any bicellular spores on the host. Later, this fungus was found to produce 2%–4% uniseptate spores on artificially inoculated plants, and hence the pathogen was renamed *A. rabiei* (Labrousse, 1931). *A. rabiei* is now the most widely adopted appellation among the scientific community despite the diverse views of taxonomists on its nomenclature. Although known for centuries, AB disease of chickpea was first formally described in the scientific literature in 1911 from the North‐West Frontier Province of India (now in Pakistan) (Butler, 1918). AB disease results in yield losses ranging from 10% to 100% under conducive environmental conditions (Knights & Hobson, 2016; Nene et al., 1981; Sharma & Ghosh, 2016; Singh, 1990). The disease manifests as necrotic lesions in a cool and humid environment and infects all the aerial parts of the host plant. Moderate to severe AB infections occur in the temperature range of 10–25°C and a wetness period of >12 h (Trapero‐Casas & Kaiser, 1992a). AB has been reported in more than 40 chickpea‐growing countries, leading to severe economic loss in the global pulse market (Galdames & Mera, 2003; Harveson, 2002; Kaiser et al., 2000; Manjunatha et al., 2018; Pande et al., 2005; Viotti et al., 2012).

The continual evolution of the pathogen makes genetic resistance and fungicide application unsustainable of themselves (Sharma & Ghosh, 2016). A thorough understanding of disease ecology and pathogen biology will assist scientists in identifying new resistance sources and minimizing the selection pressure on the pathogen. The available draft genome sequences of two aggressive *A*. *rabiei* isolates will provide a reference sequence for diversity analysis by next‐generation genotyping, associate polymorphism within the evolved isolates with aggressiveness on the host, and help in understanding *A*. *rabiei* biology through molecular studies (Shah et al., 2020; Verma et al., 2016). The purpose of this review is to provide a comprehensive profile of the pathogen with special emphasis on recent genomic and molecular studies.

## 2 LIFE CYCLE AND BLIGHT DISEASE SYMPTOMS

**FIGURE 1:**
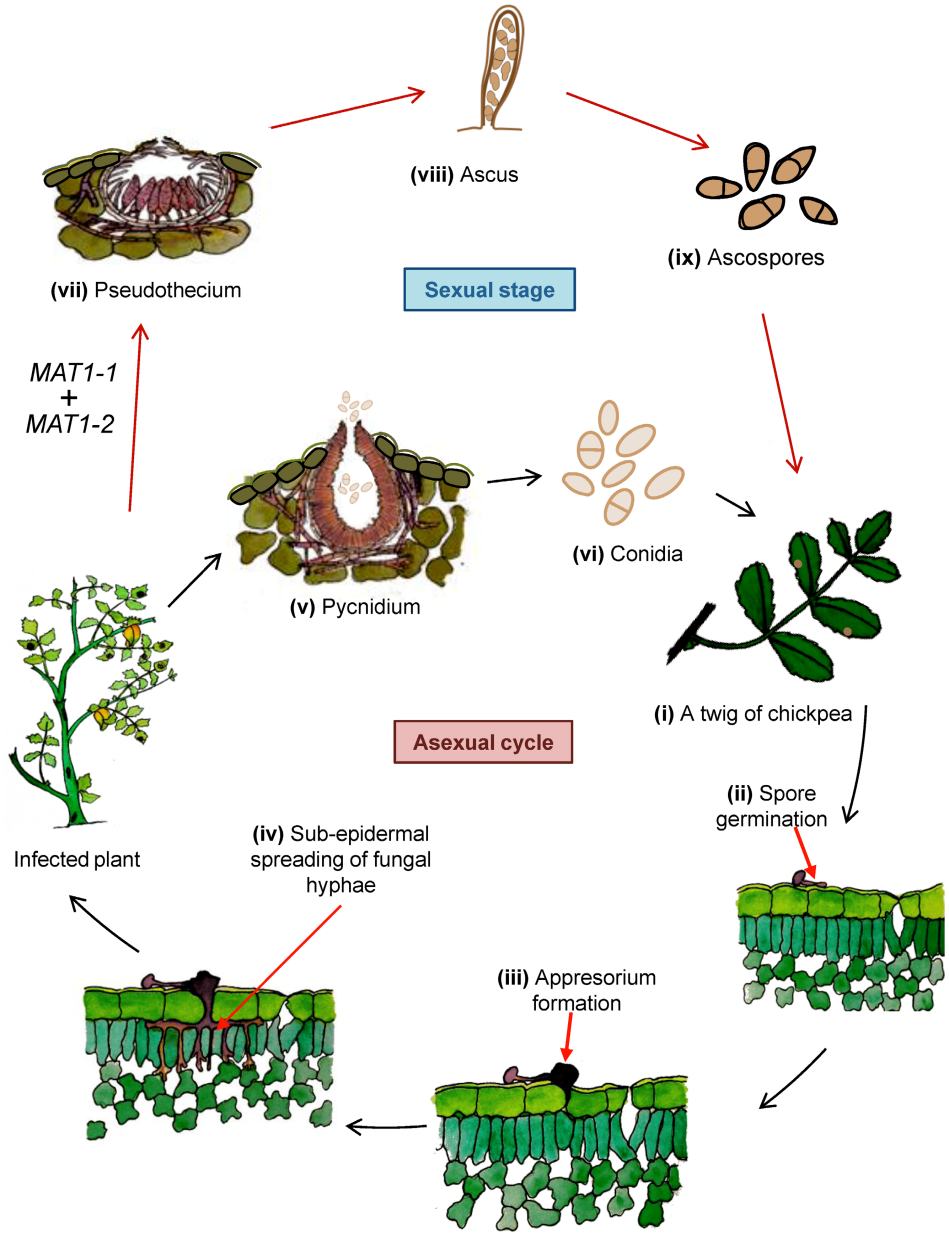
Disease progression of *Ascochyta rabiei*: sexual and asexual stages in the life cycle. (i) The airborne fungal conidia infect a chickpea plant. (ii) Conidia land on the leaf surface of the chickpea plant and (iii) start germinating through the formation of germ tubes. Later on, the germ tubes form an appressorium‐like structure at the tip of hyphae. (iv) The appressorium punctures the epidermal layer and (v) invades the subepidermal tissues. (vi) At a later stage pycnidia are formed, which contain asexual conidial spores that are dispersed by rain splash. (vii) During the sexual cycle, a specialized structure called the pseudothecium is formed on the infected plant in moist and cool conditions (winter). The pseudothecium is a cup‐like structure with an inner fertile layer called the hymen and an outer narrow opening called the ostiole. (viii) The hymen gives rise to sac‐like structures called asci that each contain eight ascospores. (ix) In spring, the pseudothecium forcefully discharges ascospores, which are carried by wind over distances of up to 10 km.

*A. rabiei* exhibits both teleomorphic (sexual) and anamorphic (asexual) stages in its life cycle. The teleomorph *D. rabiei* develops when both compatible mating types are present on AB‐infected crop debris during winter (high‐moisture and low‐temperature [5–10°C] conditions). Successful mating results in the formation of the sexual fruiting body called a pseudothecium, which is initially immersed in host tissue (Figure 1). Trapero‐Casas and Kaiser (1992b) have extensively discussed the conditions of pseudothecium development on artificially infested chickpea straw and in field conditions. Mature pseudothecia are black/dark brown, subglobose‐shaped, 120–270‐μm structures bearing numerous cylindrical to subclavate‐shaped asci. Each ascus contains eight two‐celled ascospores. Under moist conditions, mature pseudothecia forcibly discharge ascospores into the air. During sexual reproduction, meiotic recombination generates new pathogen variants and ascospores help in the long‐distance dispersal of the pathogen (Bayraktar et al., 2007; Crociara et al., 2021; Navas‐Cortés et al., 1995).

*A*. *rabiei* is heterothallic, having mating types *MAT1*‐*1* and *MAT1*‐*2*. Prior to the PCR‐based identification of mating types, laboratory crossings of unknown isolates were performed with designated mating‐type tester strains: USDA tester isolates AR‐483 (*MAT1*‐*1*) and AR‐158 (*MAT1*‐*2*) (Armstrong et al., 2001; Trapero‐Casas & Kaiser, 1992b). When the multiplex‐PCR system was developed for *A*. *rabiei* to differentiate between mating types, isolates containing an alpha (α) domain and an HMG domain at the *MAT* locus were renamed as *MAT1*‐*1* and *MAT1*‐*2*, respectively, to confirm to the nomenclature of mating type genes (Barve et al., 2003; Phan et al., 2003a). Thus, the traditionally named *MAT1‐1* and *MAT1‐2* types are now referred to as *MAT1‐1* (AR‐483) and *MAT1‐2* (AR‐158), respectively. The presence of both mating types has been confirmed in Algeria, Argentina, Bulgaria, Canada, Egypt, Greece, Iran, Israel, Italy, Libya, Morocco, Pakistan, Portugal, Spain, Syria, Tunisia, Turkey, and the USA (Pacific Northwest), but their mating‐type distribution frequency differs (Bencheqroun et al., 2022; Crociara et al., 2020; Kaiser & Küsmenoglu, 1997; Manjunatha et al., 2018). In some countries, the *A*. *rabiei* populations have a 1:1 ratio for the two mating types (Nourollahi et al., 2011; Peever et al., 2004), while others do not have an equal mating type ratio (Ali et al., 2012; Atik et al., 2011; Getaneh et al., 2021; Rhaiem et al., 2007, 2008).

Although highly aggressive isolates are regularly reported in India and Australia, the collected isolates are of *MAT1*‐*2* type only, suggesting an asexual mode of reproduction in these areas (Galloway & Macleod, 2003; Leo et al., 2015). The asexual stage of *A*. *rabiei* is characterized by the formation of an asexual fruiting body termed the pycnidium, which bears a large number of asexual spores or conidia. Conidia are hyaline, oblong to oval in shape, straight or slightly bent at both ends, and measure 6–12 × 4–6 μm (Nene, 1982). The life cycle of *A. rabiei* is schematically represented in Figure 1.

### 2.1 Source of inoculum

Contaminated seeds and ascospores serve as a major source of primary inoculum (Dey & Singh, 1994; Kimber et al., 2007). *A. rabiei* survives 13 years on seeds stored at 4°C (Kaiser, 1997) and 5 months at 10–35°C (Singh et al., 1995). Notably, infected seeds result in a low germination rate and if germinated cause early and severe disease (Iqbal et al., 2002; Kaiser, 1997; Wise, Henson, et al., 2009). *A. rabiei* also survives 8 months on infected chickpea debris within a 10–35°C temperature range (Nene & Reddy, 1987) and 20 months on infected stems (Kaiser & Hannan, 1987; Shahid et al., 2008). Pseudothecia formed on chickpea‐infected debris harbour ascospores that are carried over long distances by wind and can infect plants at a distance of several kilometres; thus, the chances of spreading the disease to new regions also increases when *A*. *rabiei* reproduces sexually (Chilvers et al., 2007; Pande et al., 2005). Ascospores germinate more rapidly than conidia (Trapero‐Casas et al., 1996; Trapero‐Casas & Kaiser, 1992a, 2007). During the growing season, conidia formed in pycnidia are dispersed locally by rain splashing, serving as secondary inoculum (Nene, 1982). Therefore, disease spread mainly occurs through the anthropogenic movement of seeds, dissemination of spores via wind and water, and infected chickpea debris. *A*. *rabiei* is known to be pathogenic to some other legumes (cowpea, lentil, common bean) after artificial inoculation and it was isolated from plants of other genera such as *Brassica nigra*, *Triticum aestivum*, *Lamium amplexicaule*, *Galium apanine*, and *Descurainia sophia* grown in fields previously containing AB‐infested chickpeas (Khan et al., 1999; Pande et al., 2005; Tivoli & Banniza, 2007). Hence, alternate *A*. *rabiei*‐infected hosts can also serve as a source of inoculum for AB disease in chickpea.

### 2.2 Mode of pathogenesis

**FIGURE 2:**
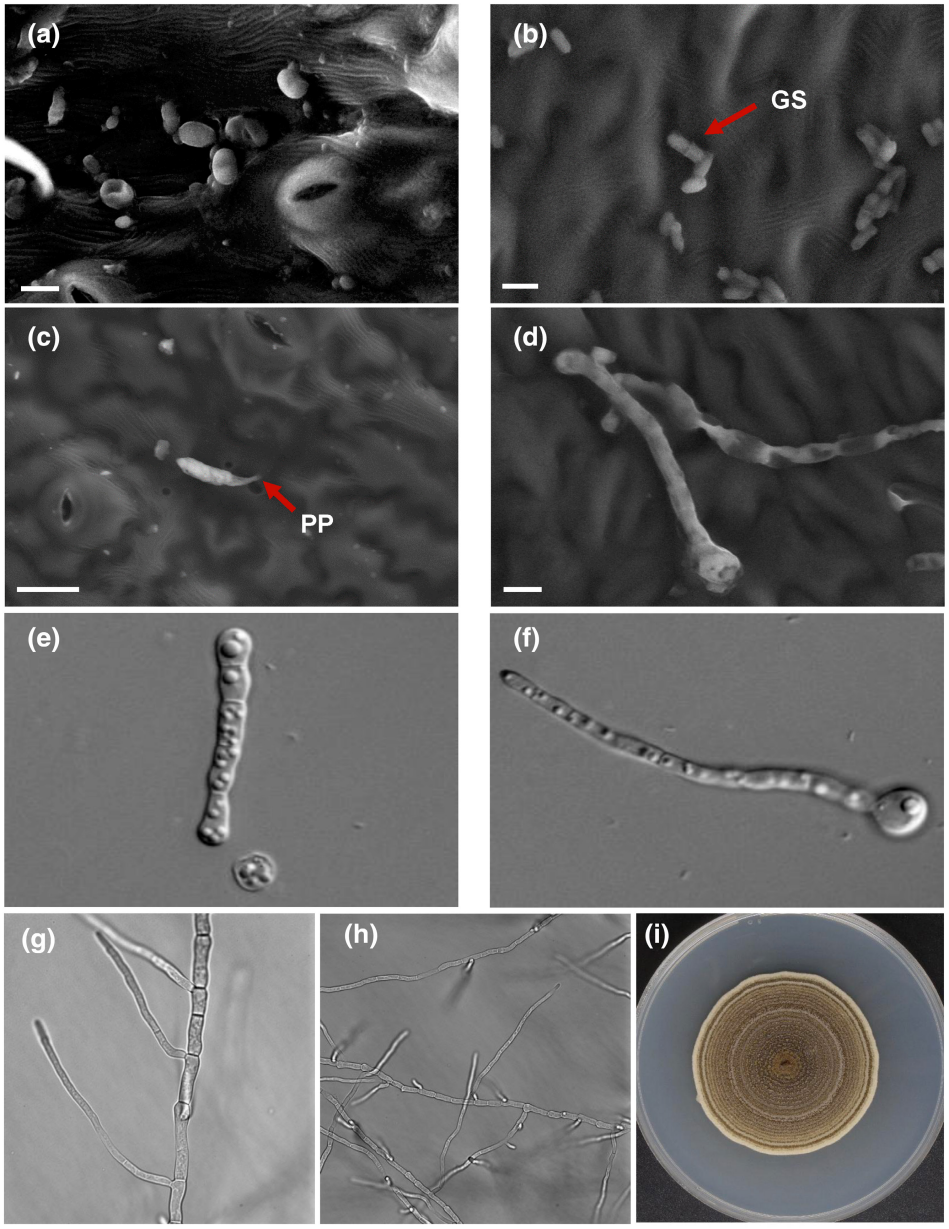
Morphology of *Ascochyta rabiei*. **(**a–d) Scanning electron and (e–i) light microscopy images. (a) Fungal spores on a susceptible chickpea leaf; bar = 10 μm. (b) Germinating spore (GS) with germ tube; bar = 10 μm. (c) Penetration peg (PP) formation; bar = 20 μm. (d) Fully grown fungal hyphae on chickpea leaves; bar = 10 μm. (e,f) Early stages of spore germination and germ tube formation observed on a glass slide. (g,h) Fungus showing hyphal branching after 2 and 3 days of growth. (i) *A. rabiei* growing on potato dextrose agar supplemented with chickpea meal.

Spores of *A. rabiei* land on the host surface and start germination via the formation of a germ tube within 12–24 h (Pandey et al., 1987). These germ tubes form appressoria and at the time of appressorium formation, mucilaginous exudates are secreted from the fungus to establish tight contact with the host and also to protect the conidia from desiccation (Höhl et al., 1990). Various stages of fungal spore germination, growth, and host invasion are shown in Figure 2(a–i). However, the germination and invasion of spores vary according to *A. rabiei* aggressiveness. Highly aggressive isolates germinate and invade quickly compared to less aggressive isolates (Sambasivam et al., 2020). *A. rabiei* isolates generally develop appressoria by 30–40 h after infection; however, on tolerant varieties, appressorium formation is delayed to 60–70 h, and also only a few appressorium are observed. Furthermore, *A. rabiei* secretes cell wall‐degrading enzymes (CWDEs) to breach the host barrier. Predominantly, *A. rabiei* shows direct penetration through the cuticle but in some cases penetration is seen via stomata (Ilarslan & Dolar, 2002). Fungal penetration occurs at the juncture of epidermal cells (Pandey et al., 1987). After penetration, fungal hyphae expand subepidermally, proceed through the middle lamella from leaf to petiole, and thus reach the stem. Subsequently, the pathogen colonizes the phloem tissues and severely damages the parenchymatous cells. However, the integrity of the xylem remains intact, albeit with some damage (Ilarslan & Dolar, 2002). Histological studies of the *A. rabiei–*chickpea pathosystem show that as the fungus expands subepidermally, the cells of the chickpea become deformed and disintegrated (Ilarslan & Dolar, 2002), indicating the secretion of phytotoxins by the fungus. Previously, cutinase (Tenhaken et al., 1997), xylanase (Bruns, 1999; Jayakumar et al., 2005), and exopolygalacturonase (pectinase) (Tenhaken & Barz, 1991) enzymes have been identified in *A. rabiei*. It was also reported that *A. rabiei* secretes solanopyrone A, solanopyrone B, solanopyrone C, cytochalasin D, and a proteinaceous toxin during pathogenesis (Chen & Strange, 1994; Hamid & Strange, 2000; Höhl et al., 1991; Kaur, 1995; Kim et al., 2017; Latif et al., 1993).

**FIGURE 3:**
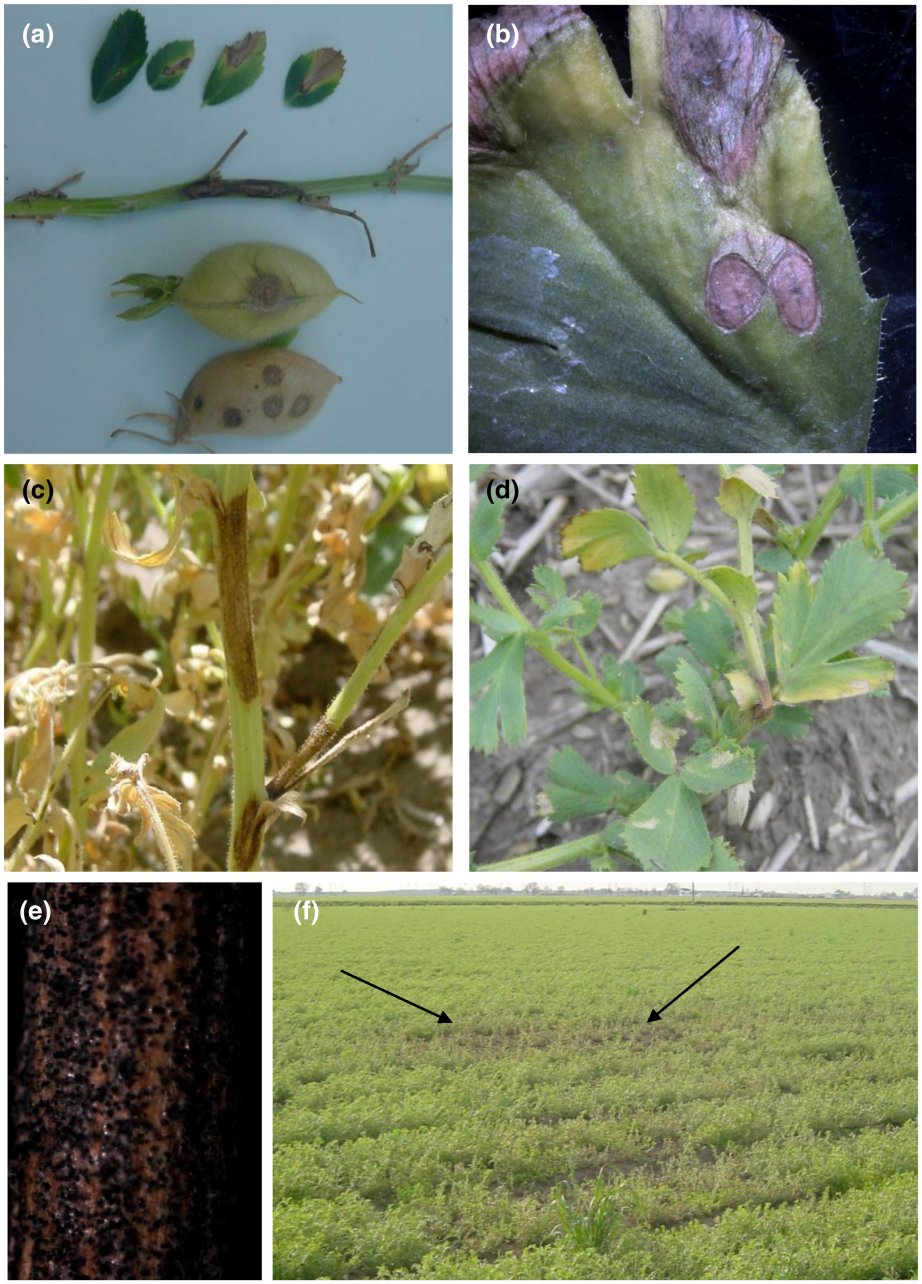
Symptoms of Ascochyta blight on chickpea. (a) Circular brown lesions on leaves and pods and elongated/oval‐shaped necrotic spots on the stem. (b) Enlarged view of lesions on the leaf. The circular lesions formed on leaves bear concentric rings of pycnidia, which is the most distinctive diagnostic feature of the disease. (c) Infected plant in the field showing severe symptoms on the stem. (d) At later stages, lesions girdle the stem and the area above the girdle falls off. (e) Debris having both pycnidia and pseudothecia. (f) Blighted patch of chickpea in the field.

Early symptoms on the chickpea leaves include small watersoaked lesions that turn brown at later stages (Figure 3). Lesions on stems and petioles are elongated and often girdle the affected parts in later stages of the disease, leading to extensive plant death (Figure 3). At a later stage of infection, *A. rabiei* secretes several molecules to counteract the host defence machinery and starts forming pycnidia, the anamorph stage fruiting bodies harbouring conidia. The oval to circular lesions formed on leaves and pods have concentric rings of pycnidia, which is the most distinctive diagnostic feature of the disease (Akem, 1999). Infection can advance from the pod to the seeds, though sometimes severely infected pods fail to produce seeds. Infected seeds appear discoloured and shrivelled and often harbour lesions bearing dark pycnidia (Pande et al., 2005). Conidia are further able to cause secondary infection in chickpeas. AB disease is more prevalent during the flowering and podding stages of the plant, although AB can appear any time after plant emergence from soil (Sharma & Ghosh, 2016). In the field, the disease manifests as patches of blighted plants that rapidly spread throughout the crop under conditions favourable to the disease (Pande et al., 2005).

## 3 DIAGNOSIS

Field diagnosis of AB disease on chickpea plants is traditionally based on symptoms. Under laboratory conditions, suspected infected plant tissue or seeds are surface‐disinfected and placed in artificial media plates (potato dextrose agar/chickpea seed meal dextrose agar/malt extract agar; Kharbanda & Bernier, 1980). After incubation (21°C, 12/12 h light/dark cycle for 23 days), the fungus is identified based on the morphological characteristics of the mycelium, pycnidia, and shape or size of conidia along with colony colour, growth rate, and conidiomatal colour via microscopy analysis (Bahr et al., 2016; Crociara et al., 2021). The field method relies on visible symptoms formed later in the infection process, and with this method AB disease can be detected at the mid to late stage of infection. Identifying the pathogen before the visibility of the symptoms is a better strategy for timely management of the disease. Visual symptom‐based identification requires abundant experience, while microscopic diagnosis requires a skilled pathologist to reliably identify the pathogen. Because the traditional method of AB identification is time consuming, rapid and unambiguous identification techniques are being developed for various applications.

The shortcomings of the *A*. *rabiei* morphological diagnosis can be overcome by molecular methods such as PCR‐based amplification of fungal diagnostic sequences. These technologies are mainly important for the diagnosis of latent *A. rabiei* infections in chickpea seeds and plant tissues where the pathogen biomass is low. To distinguish *A. rabiei* from other *Ascochyta* spp., a diagnostic PCR‐restriction fragment length polymorphism test was developed by Phan et al. (2002) which is based on PCR amplification of internal transcribed spacer (ITS) regions of *Ascochyta* species, restriction digestion of the amplified product with the enzymes *Sau*96I and *Nla*IV, and visualization of discrete banding patterns to distinguish *A. rabiei* from isolates of species such as *A. pinodes*, *A. lentis, A. fabae*, and eight other chickpea pathogens (Phan et al., 2002). The method is sensitive enough to amplify 0.1 pg of purified fungal DNA and DNA extracted from infected seed diluted up to 10^−5^. Phan et al. (2002) also described a protocol to efficiently detect fungal infections in chickpea seeds. The *HMG* regions of mating type *MAT1*‐*2* in *Ascochyta* spp. are substantially variable; hence, PCR amplification and sequencing of this region can be used to confidently diagnose blight diseases in legumes (Barve et al., 2003). A sequence‐characterized amplified region marker developed by Baite et al. (2020) was specific enough to differentiate *A*. *rabiei* from eight other chickpea fungal pathogens. This method is sensitive enough to amplify 196‐bp amplicons using conventional PCR and real‐time PCR from 0.5 ng and 1.0 pg genomic DNA, respectively. Similarly, the TP6‐F/TP9‐R primer pair from the β‐tubulin gene can differentiate *A*. *rabiei* from 10 other fungal species including common pathogens of chickpea using the traditional PCR method (Valetti et al., 2021). The regular PCR techniques are practical for normal laboratory diagnosis of *A*. *rabiei* but impractical for field diagnostics, especially in developing countries where basic infrastructure for pathogen diagnosis may be inaccessible. Loop‐mediated isothermal amplification (LAMP) techniques are used to amplify DNA efficiently, rapidly, and with high specificity under isothermal conditions and are more suitable for diagnostics in field conditions, as they require minimal expertise in molecular biology. LAMP techniques only require a DNA extraction kit, PCR chemicals, and a water bath for pathogen diagnosis. LAMP primers based on the *A*. *rabiei* ITS sequence were designed, and the lowest detectable fungal genomic DNA concentration is 6.01 × 10^−6^ ng/μl and 6.01 × 10^−4^ ng/μl under a laboratory setup (Chen et al., 2016). Furthermore, results can be visualized with the naked eye due to the change in colour of SYBR Green I dye (in tubes containing *A. rabiei*, the colour changes from gold to green). However, the primer specificity for this particular LAMP assay is unknown and remains to be validated.

Several other techniques with improved speed, accuracy, and sensitivity have been developed for the diagnosis of pathogenic organisms. However, their application in identifying *A*. *rabiei* is yet to be tested to overcome the challenges of quick sample processing, biological interference from plant molecules, other yet unrealized situations, and diagnosis costs. Recombinase polymerase amplification (RPA), an isothermal amplification‐based technique, is sensitive and highly suitable for pathogen detection in field conditions as it can be applied on various types of samples, it can be integrated with different detection strategies such as field‐applicable dipsticks, and lyophilized reagents of RPA display exceptional stability at ambient temperatures (Lobato & O'Sullivan, 2018). The RPA assay has been used for the diagnosis of *Phytophthora* species (Dai et al., 2019; McCoy et al., 2020), the bacterium “*Candidatus* Phytoplasma oryzae” (Wambua et al., 2017), and the wheat root rot‐ and spot blotch‐causing fungal pathogen *Bipolaris sorokiniana* (Zhao et al., 2021). However, to date, it has not been used for *A. rabiei* detection. Some other technologies like the Luminex PCR system and the Luminex xMAP system as an alternative to ELISA, the multispectral vision system to differentiate healthy from infected seeds, magnetic‐capture hybridization PCR, and BIO‐PCR also hold great promise in detecting fungal pathogens and possibly can be used for *A*. *rabiei* as well (Hariharan & Prasannath, 2021; Mancini et al., 2016). Two recent reviews have also highlighted the utility of the latest technologies—molecular capture probes targeting specific DNA sequences of the pathogen, and advanced biosensors—for the diagnosis of *Botrytis* species and other plant pathogens (Bilkiss et al., 2019; Dyussembayev et al., 2021). The handheld MinION sequencing system (Oxford Nanopore Technologies) can rapidly detect multiple pathogens simultaneously and sequencing data can reveal the pathogen's taxonomic status, but its analysis requires trained experts. The integration of nanotechnology with biomolecular analyses will improve the portability and versatility of pathogen diagnosis systems, which will help in informed disease management.

## 4 GENETIC DIVERSITY, POPULATION STRUCTURE, AND EMERGENCE OF AGGRESSIVE ISOLATES

The possibility of aggressive *A*. *rabiei* isolates being introduced to new regions/countries always remains a challenge despite quarantine measures because chickpea is heavily traded among countries and not all countries rigorously check imported seeds. The existing *A. rabiei* variants can also evolve into more aggressive, climate‐adaptable, and fungicide‐resistant variants. Knowledge about the pathogenic variability and the amount of genetic variation among individual isolates of a country can support the success of germplasm resistance screening programmes, germplasm conservation, chickpea breeding, and prediction of the evolutionary potential of *A*. *rabiei* (McDonald & Linde, 2002). The genetic diversity of *A*. *rabiei* isolates has been assessed using several types of DNA markers, including random amplified polymorphic DNA markers (on 53 isolates from Syria and Lebanon; Udupa et al., 1998), sequence‐tagged microsatellite site (STMS) markers (on 36 Australian isolates; Phan et al., 2003b), amplified fragment length polymorphism, simple‐sequence repeat (SSR) markers (on 64 isolates from the Northwest Plain zone of India; Varshney et al., 2009), and rep‐PCR markers (on 29 isolates from Iran; Azizpour & Rouhrazi, 2017). However, this limited number of markers on a low number of isolates does not illustrate the true picture of *A*. *rabiei* diversity and the population structures of a region/country as these markers are not uniformly distributed over the genome.

Among 598 *A*. *rabiei* isolates from Australia, 17% were highly aggressive, and grouping based on SSR genotyping showed that the majority of them belonged to a single dominant haplotype, ARH01 (Mehmood et al., 2017). This points toward the evolution of *A*. *rabiei* “super‐isolates” within haplotype ARH01 to proliferate in AB‐resistant cultivars and adapt to diverse agrogeographical environments within Australia. However, the diversity and structure analysis of the *A*. *rabiei* population using next‐generation genotyping later showed that SSRs had a limited ability for genotyping (Guichoux et al., 2011). The 279 isolates collected during 2013–2018 from Australian chickpea‐growing regions were genotyped using the DArTseq approach and the genotype–phenotype associations were deduced (Bar et al., 2021). Here, isolates belonging to haplotype ARH01 clustered into various groups. Furthermore, a genome‐wide association study (GWAS) revealed two single‐nucleotide polymorphisms (SNPs) that were significantly associated with the pathogenicity levels of *A*. *rabiei*.

Inoculum build‐up from minimal to no crop rotation leads to the emergence of new pathogenic forms in *A*. *rabiei* (Ford et al., 2018). Also, the genotypic diversity of outcrossing pathogenic fungal populations may allow them to overcome host resistance, so checking the mating‐type distribution and frequency can help in predicting future disease patterns. Nevertheless, *A*. *rabiei* populations exhibiting only asexual reproduction have also overcome host resistance (Bedi & Athwal, 1962; Mehmood et al., 2017; Nene et al., 1982; Sambasivam et al., 2020). The evolution of aggressive isolates has led to higher susceptibility in formerly AB‐resistant cultivars such as F8, C1234 (resistant until 1950–1951), and C235 (recommended in 1962) (Bedi & Athwal, 1962; Nene et al., 1982). Pathogenic variability of *A. rabiei* was first reported in India in 1969 (Katiyar & Sood, 1985). A new highly aggressive isolate, classified as pathotype IV, was reported from the Kaljebrine area of Syria and it was able to infect all four chickpea differential lines, namely ILC1929, ILC482, ILC3279, and ICC12004 (Imtiaz et al., 2011). In Australia, it was found that newly emerging aggressive isolates have overcome the resistance of PBA HatTrick, Genesis 090, and other cultivars (Mehmood et al., 2017; Sambasivam et al., 2020). In 2017, AB disease caused heavy losses of cultivar PBA Seamer.

As suggested above, SSR markers are labour‐intensive and provide very little information in *A*. *rabiei* as compared to the uniformly distributed SNP markers. Therefore, future studies should use next‐generation genotyping methods and a collection of a large number of isolates to illustrate the true picture of a country's *A*. *rabiei* populations and diversity. Also, emphasis should be given to gathering reliable pathogenicity or aggressiveness data for collected isolates. The practical guidance and best practices put forward by Grünwald et al. (2017) need to be followed for population genetic analysis in phytopathogens so that a useful inference of genetic diversity and population structure can be derived for sustainable disease management.

## 5 MOLECULAR STUDIES

### 5.1 Draft genome assemblies of two aggressive isolates

Genomic studies of many phytopathogens gained pace after the emergence of next‐generation sequencing (Kumar & Verma, 2012). The draft genomes of *A*. *rabiei* isolates from India (ArD2; ITCC4638; D‐11 of Singh & Pal, 1993) and Australia (ArME14, collected at Medina in 2004) were assembled after generating Illumina short‐reads and a hybrid of Illumina and PacBio reads, respectively (Shah et al., 2020; Verma et al., 2016). The ArME14 genome assembly (40,927,385 bp) is 6.26 Mb longer than the ArD2 assembly, while the genomes harbour 11,257 (ArME14) and 10,596 (ArD2) predicted protein‐coding genes. This difference in gene number is possibly due to the use of different prediction methods and sequencing technologies or due to the independent evolution of isolates. The ArME14 genome has at least 17 chromosomes, among which nine have been fully sequenced. The ArME14 mitochondrial genome “Mitochondrion MT” was also separately assembled (Shah et al., 2020). Akamatsu et al. (2012) estimated the *A*. *rabiei* genome size in the range of 23–34 Mb through electrophoretic karyotyping and suggested the presence of 12–16 chromosomes.

Small secreted virulence effectors suppress immunity of the host and subvert metabolism. This makes the initial phase of infection by biotrophs and necrotrophs similar (Verma et al., 2016; Zhu et al., 2013). The Indian group of researchers developed a pipeline that predicted 758 potentially secreted proteins in ArD2. SignalP v. 5.0 predicted 1145 secreted proteins in ArME14; however, in ArD2, the prediction was 1111 secreted proteins (Shah et al., 2020). When EffectorP v. 2.0 was implemented on both genomes, comparable numbers of effectors were predicted, namely, 36 in ArD2 and 39 in ArME14. The presence and absence of different effectors in both isolates' genomes should be verified by PCR and their functions should be investigated through gene knockout experiments.

### 5.2 Transcriptomic studies

To invade hosts and use host components for their growth, fungi secrete an arsenal of CWDEs, host‐selective toxins, and other virulence factors. In the chickpea–*Ascochyta* interaction, *A. rabiei* must produce various antioxidants to scavenge the host‐generated reactive oxygen species (ROS). To understand the molecular responses of *A. rabiei* against these stresses, Singh et al. (2012) prepared a suppression subtractive hybridization cDNA library after various oxidative stress treatments with agents such as menadione and H_2_O_2_. Menadione was used to mimic the harsh ROS conditions faced by *A*. *rabiei* in chickpeas. Superoxide dismutase, catalase, and thioredoxin genes were found to be involved in direct ROS detoxification (Singh et al., 2012). A solanopyrone biosynthesis gene cluster was identified in the AT‐rich region comprising the *Sol1*–*Sol5* genes. The *Sol5* gene encodes a diels‐alderase enzyme involved in the final step of solanopyrone biosynthesis (Kim, Park, Park, et al., 2015). Deletion of *sol5* in both *A*. *rabiei* and *Alternaria solani* showed that solanopyrones are not required for pathogenicity (Kim, Park, Park, et al., 2015). Solanopyrone A is specifically produced during saprobic growth of *A*. *rabiei* to suppress the growth of competing fungi and help in fungal survival in chickpea debris (Kim et al., 2017). The *Sol4* gene encodes a Zn(II)_2_Cy_6_ zinc cluster transcription factor (TF) and serves as a solanopyrone biosynthesis pathway regulator (Kim, Park, Gang, et al., 2015). However, in‐depth molecular analysis of *A. rabiei* genes remains to be conducted, and to date, only these few genes have been functionally characterized. Fondevilla et al. (2015) identified many crucial pathogenicity factors of *A. rabiei* using RNA sequencing and the Massive Analysis of cDNA Ends analysis tool, which was used to identify 596 transcripts that were up‐regulated during the infection. The genes highly expressed or only expressed during pathogenesis encode CWDEs, namely cellulase, pectinase, proteinases, polygalacturonase, and xylanases. Collectively, these CWDEs might help the fungi in tissue maceration and in inducing host cell death.

Recently, by using a reference‐based transcriptomics approach, 73 differentially expressed genes were identified under oxidative stress conditions (Maurya et al., 2020). These findings constitute the beginning of functional genomics of *A. rabiei* that will help in the detailed investigation of transcriptional reprogramming during infection. Our understanding of the pathogenicity factors of the fungus was further expanded by the identification of regulatory TFs crucial for the survival and virulence of *A. rabiei* (Verma et al., 2017). TFs play an critical role during host–pathogen interaction by reprogramming gene expression. A genome‐wide identification study predicted that 381 TFs are present in *A. rabiei*, among which Myb and Zn(II)_2_Cys_6_ family TFs were predominant (Verma et al., 2017). A stress‐responsive TF, ArCRZ1, has been identified as regulating the transcript levels of *ArF‐BAR*, which encodes an F‐BAR domain‐containing protein. This modulates the virulence of *A. rabiei* through effector secretion, endocytosis, and actin cytoskeleton regulation (Sinha et al., 2021). Elucidation of the expression patterns of putative effectors in resistant, tolerant, and susceptible chickpea lines at various stages of pathogen infection will help in deciphering their stage‐specific roles in virulence.

### 5.3 Genetic transformation

Genetic transformation has paved a way for the identification and functional characterization of various genes in this fungal pathogen. Protoplast isolation and polyethylene glycol‐mediated *A*. *rabiei* transformation protocols were established in 1995, albeit with low transformation efficiency (Köhler et al., 1995; Weltring et al., 1995). A stable DNA transfer method mediated by *Agrobacterium tumefaciens* using the *TrpC* promoter and a hygromycin resistance gene was later proposed (White & Chen, 2006). The transformants were confirmed with PCR and Southern hybridization indicating single integration of transfer DNA. Genes encoding fluorescently tagged proteins (EGFP and DsRED), driven by the *gpd*A promoter, have been used to visualize the early stages of fungal colonization (Nizam et al., 2010). Microscopic analysis of fluorescently tagged fungus enabled the detailed investigation of events during host–pathogen interactions.

## 6 *A. RABIEI* NEEDS A UNIFORM PATHOGENIC VARIABILITY‐BASED CLASSIFICATION

The determination of pathogenic variability among isolates of a pathogen population is important for germplasm selection and efficient resistance breeding for higher‐yielding cultivars. However, techniques for assaying disease severity during the *Cicer–A*. *rabiei* interaction and the classification of *A*. *rabiei* isolates into pathogenic variability groups are not uniform across countries with AB disease occurrence. Each research laboratory catalogues the isolates based on quantitative measurement of disease severity of a few selected chickpea differential lines and presents their isolate classification (Table 1). “Physiological races” or “races” and “pathotypes” are two commonly used terms for grouping pathogenic *A*. *rabiei* isolates based on variability, but their definitions do not fit the available information on the *Cicer–Ascochyta* interaction. Taylor and Ford (2007) critically reviewed the use of these classifications in legume–*Ascochyta* interactions. They argued that the pathogenic variability of Australian *A*. *rabiei* isolates on chickpea appears to be quantitative, where disease severity is measured on an aggressiveness scale (quantitative level of infection) and a continuum of pathogenicity appears more likely than discrete pathotypes (Elliott et al., 2011). Similar quantitative inheritance of aggressiveness was found in 99 *A*. *rabiei* isolates from Canada (Vail & Banniza, 2008). Chen et al. (2004) identified AB disease phenotypes in 48 chickpea genotypes as bimodal against pathotype I and as continuous against pathotype II isolates. Therefore, the aggressiveness of isolates must be controlled by many genes. AB disease resistance in chickpea is also quantitative, governed by few major and minor resistance‐providing genes (Labdi et al., 2013). Ideally, the grouping of isolates into pathotypes is done based on the qualitative difference in virulence on a host genotype, while knowledge about the functional resistance genes in a set of differential lines is required for the grouping of isolates into races (Taylor & Ford, 2007). Resistance gene loci within a set of chickpea differential lines can be identified using QTL‐seq, a next‐generation genotyping technique based on a forward genetic approach (Kumar et al., 2018). Also, emphasis should be given to identifying pathotype‐specific quantitative trait loci (QTLs) in chickpea.

**TABLE 1:** Classification of *Ascochyta rabiei* isolates based on pathogenic variability or aggressiveness on chickpea differential lines/cultivars

Collection site	No. of isolates	Classification	Differential chickpea lines/cultivars used	References
India		2 races and 1 biotype	5 differential lines (1‐13, EC‐26435, C‐235, F‐8, and V‐138)	Vir and Grewal (1974)
Syria and Lebanon	50	6 races	6 differential lines (ILC1929, F8, ICC1903, ILC249, ILC3279, and ICC3996)	Reddy and Kabbabeh (1985)
India (north Indian States)	348	12 races	12 differential chickpea lines	Singh (1990)
India	11	5 races	7 differential cultivars (P1343‐1, P5292‐1, C‐235, V‐138, ILC1929, ILC249, and I‐13)	Singh and Pal (1993)
USA (Palouse)	39	11 (A–K) virulence forms	15 differential lines (ILC72, ILC194, ILC202, ILC215, ILC249, ILC482, ILC1929, ILC2506, ILC3279, ICC1903, ICC3996, ICC9189, F‐85‐111, F‐85‐84, and UC‐5)	Jan and Wiese (1991)
Pakistan	102	8 virulent forms	11 chickpea differentials	Jamil et al. (1995)
Italy	41	3 pathogenicity groups	6 lines (ILC1929, ILC200, ILC482, ILC484, ILC191, and ILC3279)	Porta‐Puglia et al. (1996)
India, Pakistan, Spain, and USA	44	11 pathotypes (A–K)	7 differential lines (ILC1929, C235, ILC249, ICC1903, ILC72, ICC3996, and ILC3279)	Navas‐Cortés et al., 1998
Syria and Lebanon	53	3 pathotypes	3 differential lines (ILC1929, ILC482, and ILC3279)	Udupa et al. (1998)
India	348	12 races	12 differential genotypes	Singh and Sharma (1998)
Pakistan	130	3 pathotypes	3 differential lines (ILC1929, ILC482, and ILC3279)	Jamil et al. (2000)
USA	44	2 pathotypes	48 chickpea germplasm lines	Chen et al. (2004)
Canada (Saskatchewan)	40	14 pathotypes	8 differential lines (UC27, ICC4200, ICC4475, ICC6328, Sanford, ILC3856, FLIP83‐48, and ILC4421)	Chongo et al. (2004)
India	14	8 pathotypes	16 differential lines (ICC12, ICC607, ICC2165, ICC3918, ICC4200, ICC4475, ICC5124, ICC6306, ICC7002, ICC13754, ICC14911, ICCX810800, ICCX910028‐39ABR‐BP‐10ABR‐BP, ILC3870, FLIP 82–258, and Pb7 [ICC4991])	Basandrai et al. (2005)
Turkey	64	3 pathotypes and 6 physiological races	7 differential lines (ILC1929, F8, ICC1903, ILC249, ILC482, ILC3279, and ICC3996)	Turkkan and Dolar (2009)
Syria	10	4 pathotypes	4 differential lines (ILC1929, ILC482, ILC3279, and ICC12004)	Imtiaz et al. (2011)
Algeria (northwestern)	16	3 pathotypes and 6 physiological races	7 differential lines (ILC1929, F8, ICC1903, ILC247, ILC482, ILC3279, and ICC3996).	Benzohra et al. (2011, 2012)
Iran	30	10 virulent forms and 16 pathogenic groups	7 differential lines	Ghiai et al. (2012)
Syria	133	4 pathotypes	5 differential lines (ICC‐12004, ICC‐3996, ILC‐3279 [Ghab‐2], FLIP 82–150C [Ghab‐3], and ILC‐263)	Atik et al. (2013)
Pakistan	21	3 virulence groups	5 differential lines (AUG‐424, Pb‐1, AUG‐480, CM‐72, and Paidar)	Sarwar et al. (2013)
Iran (western provinces)	40	6 pathogenic groups	8 differential lines (ILC1929, PCH215, ILC194, ILC482, ILC3279, ICC3996, ILC72, and ILC202)	Vafaei et al. (2015)
Algeria	16	3 pathotypes	3 differential lines (ILC1929, ILC482, and ILC3279).	Mahiout et al. (2015)
Algeria	20	4 pathotypes	differential lines (ILC1929, ILC482, ILC3279, and ICC12004)	Benzohra et al. (2018)
India	25	7 races	10 differential lines (ICC11879, ICC4991, ICC3996, ICC15978, ICC1467, ICC1903, ICC1527, H00108, GL26054, and GPF2)	Baite and Dubey (2018)
Iran	32	6 races	7 differential lines (ILC1929, ILC5928, ILC202, ILC72, ICC3996, ILC194, and PCH215)	Farahani et al. (2019)
Australia	279	6 pathogenic groups	4 differential lines (ICC3996, Genesis090, HatTrick, and Kyabra)	Bar et al. (2021)

Screening of chickpea germplasms that are resistant against AB disease and identification of pathogenic variability among isolates requires globally agreed upon screening and evaluation methods. Bioassays based on these methods will help in comparing the results of one region with others and avoiding *A*. *rabiei* isolate sharing within and between countries will decrease the chances of accidental release of aggressive *A*. *rabiei* isolates to new regions. Consensus on the methods and conditions for evaluating chickpea lines and *A. rabiei* isolates in the field and under controlled conditions should be reached in upcoming legume conferences and *Ascochyta* spp. workshops where breeders and pathologists meet. A global and uniform classification of pathogenic variability can be achieved by discussing the following points:

1. Researchers should describe all the field conditions (temperatures at various time points of the day, humidity, sunlight intensity, etc.) when screening germplasms or populations for AB disease resistance mapping.
2. The culture media, incubation conditions (such as light intensity, photoperiod, and temperature), and the concentration and amount of conidial suspension used for chickpea inoculation should be mentioned.
3. The plant age, number of plants per pot, pot size, humidity under controlled conditions, light intensity, photoperiod, and temperature for vegetative and reproductive stage testing should be reported.
4. Plant inoculation and incubation procedures and conditions should be described in detail, because various protocols such as mini‐dome, cloth chamber screening, and plastic cages exist at the moment.
5. The scale used for evaluation of AB disease scores and at what point resistance or susceptibility is considered should be reported.
6. A set of differential lines to be used for AB disease resistance screening should be established so that global consistency can be maintained.

## 7 AB DISEASE MANAGEMENT

Successful management of AB disease requires integrated disease management to keep the disease below the economic threshold (Ford et al., 2018). The management practices integrate appropriate cultural practices and judicious application of fungicides. The cultural practices for AB disease management mainly include the use of available moderately resistant cultivars and a combination of practices aimed at reducing sources of pathogen inoculum levels by rotation of chickpea fields with nonhost crops, the use of healthy seeds for planting, and deep‐burying of diseased plant debris when practical (Ford et al., 2018; Gan et al., 2006; Shahid et al., 2008). The seed sowing time can also be optimized per region so as to plant after the time of ascospore release and so that the least favourable conditions for AB disease development occur during chickpea flowering. In India, a major shift in chickpea production from the northern states to the middle and southern states has reduced the incidence of AB disease due to conditions that are less suitable for AB disease to spread in these states during the winter season. Avoiding chickpea sowing in the same paddock for 3–4 years is another preventive measure; however, it is a less practical solution for farmers with small landholdings in Africa, West Asia, and the Indian subcontinent. Cultural practices and chemical treatments alone are not efficient in preventing yield loss due to AB disease (Cho et al., 2004; Manjunatha et al., 2018). Thus, the development and deployment of resistant cultivars is the most effective, economical, and environmentally safe strategy for AB disease management (Crutcher et al., 2022). These integrated disease management practices will reduce selection pressure and avoid the emergence of new pathogen races, thus averting crop loss.

### 7.1 Chemical control of AB disease

Despite the fact that fungicides are costly and potentially harmful to the environment, the only effective measure available to control AB disease during the growing season is the application of fungicides. Because of the lack of highly resistant chickpea cultivars, fungicide application is often necessary for profitable chickpea production (Pande et al., 2005). Fungicides are used in two ways: seed treatment and foliar application. Infected seed is a source of primary inoculum in addition to being a means that introduces AB disease to new production areas. Thus, seed treatment is an effective measure to reduce the inoculum level. Seed treatment cannot completely eradicate seedborne inoculum but will significantly reduce the inoculum level carried on the seed. Evidence is available that seed treatment with systemic fungicides such as strobilurins enhances plant resistance of young seedlings. Seed treatment with the fungicide thiabendazole or a combination of thiabendazole and other chemicals can prevent disease spread through contaminated seeds (Wise, Henson, et al., 2009).

Fungicides can be protectants or systemic. Protectant fungicides need to be applied before infection, whereas systemic fungicides may be applied up to 4 days postinfection. In situations of rain, the preventative application of the protectant fungicide chlorothalonil can delay the onset of the disease (Bretag et al., 2008). Three groups of systemic fungicides, namely demethylation inhibitors (DMIs), quinone outside inhibitors (QoIs), and succinate dehydrogenase inhibitors (SDHIs), are widely used in chickpea protection against AB disease. During critical periods of flowering and pod filling, application of a systemic fungicide (Proline [DMI], Endura, and Priaxor [SDHIs]) is generally advised if the conditions are favourable for AB disease. The systemic fungicide tebuconazole or difenoconazole (DMIs) was found to be effective when applied up to 3 days postinfection. It also suppressed the disease as effectively as protectant applications after rain or overhead irrigation (Shtienberg et al., 2000).

Repeated application of the same fungicides, especially single‐site mode of action fungicides, may lead to the development of fungicide insensitivity (resistance). Thus, there should be a well‐thought‐out plan for fungicide application to prevent the development of fungicide resistance in *A. rabiei* populations, taking into account the timing of application, the application rate, and rotation of fungicide (Pritchard, 2000). Among 66 single‐spore isolates, insensitivity to one or more fungicides (chlorothalonil and mancozeb [broad‐spectrum fungicides] and pyraclostrobin [QoI]) was detected in 49 isolates (Chang et al., 2007). Resistance to QoI fungicides in *A. rabiei* has been reported in chickpea‐growing areas of Canada and the USA (North Dakota and Montana) (Delgado et al., 2013; Gossen & Anderson, 2004; Owati et al., 2017; Wise, Bradley, et al., 2009). The site‐specific mode of action of QoI and SDHI (fluopyram) fungicides increases the potential for the development of insensitivity in pathogen populations; hence, these two fungicides are at higher risk of insensitivity development in pathogens. If using QoI fungicides to control AB, then local isolates should be checked for sensitivity in vitro on culture plates, by PCR with specific primer sets, and with hydrolysis probes to determine the presence of QoI‐resistant isolates (Owati et al., 2017). Currently, DMI and SDHI fungicides are the preferred fungicides in chickpea production (Kandel et al., 2020). Judicial use of these fungicides with monitoring is a prerequisite to avert the emergence of resistant isolates. Lonergan et al. (2015) checked the sensitivity of *Ascochyta* species isolates (collected from the Pacific Northwest) to the fungicides boscalid, fluxapyroxad, and prothioconazole. The discriminatory concentration (1 μg/ml) was established for *A. rabiei* and will be used to monitor sensitivity shifts and to make effective disease management recommendations. These studies have the potential to limit the outbreak of the disease and to prevent the failure of control by available fungicides as well as huge crop losses.

Judicial use of fungicides requires an accurate and timely forecast of the disease. Toward that end, many studies have aimed at developing forecast models for AB disease development and fungicide application (Diekmann, 1992; Jhorar et al., 1997; Kaur et al., 2011; Rhaiem & Cherif, 2014; Shtienberg et al., 2005). An integrated model considering weather conditions, the *A. rabiei* life cycle, and susceptibility and growth stage of the chickpea cultivar would greatly facilitate using forecasting support systems in the management of AB disease in chickpea. Recently, Salotti and Rossi (2021), using systems analysis on available data in the literature, developed a mechanistic weather‐driven model for the prediction of AB disease epidemics. Such an analysis revealed some gaps in our knowledge for future investigation. Nevertheless, their model was validated by independent data available in the literature and showed a high concordance correlation coefficient (0.947) between predicted and observed data. Available data show this model is accurate and reliable, representing a significant advance in the application of precision agriculture in terms of fungicide application in managing AB disease.

As alternatives to chemical fungicides in managing AB diseases, some biological products have been tested, such as the *n*‐hexane extract fractions (dried and powdered plant samples were dissolved in *n*‐hexane) of *Chenopodium album* and *Syzygium cumini* (Jabeen & Javaid, 2010; Sherazi et al., 2016), ethyl acetate and *n*‐butanol fractions of *Withania somnifera* and *Tagetes erectus* extracts (Javaid et al., 2020; Shafique et al., 2011), and essential oils of oregano (*Origanum compactum*), thyme (*Thymus vulgaris*), peppermint (*Mentha piperita*) and lemon‐scented gum (*Corymbia citriodora*) (Ennouri et al., 2020; Erdogan & Keceli, 2021). These studies were generally conducted under laboratory conditions and some did show significant inhibition of *A. rabiei* in vitro. However, the efficacy of these antifungal compounds still needs to be tested in field conditions.

### 7.2 AB‐resistant germplasm, QTL mapping, and breeding for AB resistance

Regular screening of diverse chickpea germplasms for resistance against new aggressive *A*. *rabiei* isolates under different environments and introgression of AB resistance into higher‐yielding cultivars is required for effective AB disease management (Pande et al., 2005, 2010). Previously, large‐scale screening of *kabuli* and *desi*‐type chickpea germplasm against *A. rabiei* was performed at ICARDA, Syria (Reddy & Singh, 1984; Singh & Reddy, 1992, 1993a). A multilocation screening of 112 lines in 11 countries revealed four suitable lines (ILC72, ILC191, ILC3279, and ILC3856) that were AB‐resistant in most countries (Singh et al., 1984). As a result, these lines and their derivatives were widely used as AB‐resistant parents for developing mapping populations and resistance breeding. Only three accessions (ICC1915, ICC7184, and ICC11284) were AB‐resistant among ICRISAT's mini‐core collection of 211 chickpea accessions, which is 1.1% of accessions of the entire ICRISAT chickpea collection (Pande, Kishore, et al., 2006). Recently, five promising AB‐resistant accessions were identified from a total of 1970 chickpea accessions (originating from 17 countries) screened during multiple seasons at two locations in India (Gayancharan Rani et al., 2020). In wild *Cicer* species, out of 201 accessions, 4 *C. judaicum* and 7 *C*. *pinnatifidum* accessions were found to be resistant or moderately resistant to AB disease (Singh & Reddy, 1993b). In another study on 148 *Cicer* accessions, 5 resistant and 55 moderately resistant accessions were found (Pande, Ramgopal, et al., 2006).

New AB‐resistant chickpea accessions are regularly identified globally, but resistance breeding without molecular markers has noticeably decreased the diversity in new cultivars. This decrease in diversity is due to linkage drag around the genomic regions where AB resistance genes are present. This is noticeable in Australian genotypes, mainly released for AB resistance, where chickpea chromosome 4 (Ca4) has a lower level of genetic diversity (Li et al., 2017). The AB resistance locus mapping on chickpea genetic maps and its marker‐assisted introgression into farmers' adapted cultivars required polymorphic DNA markers (Millan et al., 2010; Winter et al., 1999). After the development of STMS markers, most of the AB resistance‐associated QTLs were mapped on chickpea linkage group 4 (LG4) and other linkage groups (Table 2). The development of chickpea's integrated physical, genetic, and genome map also contributed to more recent studies (Varshney, Mir, et al., 2014). Two GWAS on 69 chickpea genotypes and 132 advanced lines unveiled the AB4.1 genomic region (a cluster of 20 SNPs) and one SNP, respectively, significantly associated with chickpea resistance against the Australian *A*. *rabiei* isolate FT13092‐1 (Li et al., 2017). The SNP's genomic position also overlaps with the AB_echino QTL, recently identified as being involved in AB resistance in an interspecific cross‐derived population (Sudheesh et al., 2021). Newman et al. (2021) performed GWAS on 138 *Cicer reticulatum* accessions and identified four SNPs associated with AB resistance against ME14 and 15CUR005 isolates of *A. rabiei*. Few previous QTL mapping studies have mentioned *A*. *rabiei* isolates and their pathotypes: pathotypes I and II (Cho et al., 2004; Udupa & Baum, 2003), pathotype II (Taran et al., 2007), and pathotype III (Taleei et al., 2010). The chickpea AB differential lines set should be used for disease phenotyping under field and controlled conditions of a new chickpea population. The differential lines used in various studies are listed in Table 2. This helps in comparing the pathogenicity or aggressiveness of the *A*. *rabiei* isolate used on a new chickpea population and differential lines set in that particular environment. It will help in identifying QTLs working against a pathogenic group and will greatly help breeders in planning their strategies for AB resistance breeding for high‐yielding local environment‐adapted varieties. The application of newer fast‐forward genetic methods (QTL‐seq, mQTL‐seq, MutMap, etc.) will reduce the time required for QTL fine‐mapping and robust marker development in newer AB‐resistant lines (Nguyen et al., 2019; Singh et al., 2022).

**TABLE 2:** Genetic studies to identify linkage groups (LGs) and quantitative trait loci (QTLs) for Ascochyta blight resistance using intra‐ and interspecific biparental populations

Resistant parent	Susceptible parent	LGs and QTLs	Flanking markers	References
FLIP84‐92C	PI599072 (*Cicer reticulatum*)	LG1: QTL2 LG4: QTL3 LG6 (**now LG4**): QTL1	QTL1: UBC733b ‐ UBC181a QTL2: UBC836b ‐ Dia4 QTL3: UBC681a ‐ UBC858b	Santra et al. (2000)
FLIP84‐92C (2)	PI359075(1)	LG4	QTL1: GAA47 QTL2: TA72, GA2	Tekeoglu et al. (2002)
ICC12004	Lasseter	LG1: QTL1 LG2: QTL2, QTL3 LG3 (**now LG4**): QTL4, QTL5, QTL6	QTL1: STMS28‐TS12b QTL2: TA3a‐TS45 QTL3: TS45‐TA3b QTL 4: PTOFENb212‐TA130 QTL5: TA130‐TA146 QTL 6: TA146‐ClRRinn904	Flandez‐Galvez et al. (2003)
ILC3279	ILC1272	LG2: ar1, ar2a LG4: ar2b	ar1: GA16 ar2a: GA16 ar2b: TA130, TA72, TS72	Udupa and Baum (2003)
PI527930 (*Cicer echinospermum*)	Lasseter	LG4: QTL1, QTL2	QTL1: STMS11, GA2, TR20 QTL2: XLRRb280	Collard et al. (2003)
ILC3279	CA2156	LG4: AR2	SC/OPK13603,	Millan et al. (2003)
ICC4958	PI489777	LG4: qtl1	STMS11, GA2, GAA47, TR20	Rakshit et al. (2003)
FLIP84‐92C(2)	PI359075(1)	LG2A + 6B: Ar19 LG2B: Ar21d LG4A	Ar19: GA16 Ar21d: TA37‐TA200 LG4A: GA24‐GAA47	Cho et al. (2004)
ILC3279	WR315	LG4a: QTL_AR1_ LG4b: QTL_AR2_	QTL_AR1_: B/b‐UBC881_465_‐GAA47 QTL_AR2_: TA146‐SCY17_590_	Iruela et al. (2006)
Hadas	ICC5810	LG4: QTL4.1, QTL4.2 LG8: QTL8	QTL4.1: H3C041, TA2 QTL4.2: H1A12/H1H13, H1G20 QTL8: TA3 and H3C11a	Lichtenzveig et al. (2006)
ILC72	Cr5‐10	LG2	UBC881_621_ and OPAI09_746_	Cobos et al. (2006)
CDC Frontier	ICCV96029	LG3: QTL1 LG4: QTL2 LG6: QTL3	QTL1: TA64 QTL2: TS54 QTL3: TA176	Taran et al. (2007)
ILC3279	WR315	LG2: QTL_AR3_	QTL_AR3_: TR58, TA194, TS82	Iruela et al. (2007)
FLIP84‐92C	PI599072	LG4: QTL1	20(T)l12‐Right	Rajesh and Muehlbauer (2008)
ICCV04516	ICC4991 (Pb7)	LG3: QTL1 LG4: QTL2, QTL3	QTL1: TR58 QTL2: TA146‐ TR20 QTL3: TA2, TAA170	Kottapalli et al. (2009)
CDC Frontier	ICCV96029	LG3: QTL2 LG4: QTL3 LG6: QTL4	QTL2: TA64 QTL3: TS54 QTL4: TA176	Anbessa et al. (2009)
CDC Luna	ICCV96029	LG2: QTL1 LG4: QTL3	QTL1: TR19 QTL3: TS54	
CDC Corinne	ICCV96029	LG4: QTL3 LG8: QTL5	QTL3: TA132 QTL5: TS45	
Amit	ICCV96029	LG3: QTL2	QTL2: TA64	
ICC3996	ILWC184 (*C. reticulatum*)	LG3: QTL3[9] LG4: QTL4[1], QTL4[7]	QTL3[9]: TA34‐TA142 QTL4[1]: STMS11‐TAA170 QTL4[7]: H1A12‐H3D09	Aryamanesh et al. (2010)
ICC12004	Bivanij	LG3, LG4, LG6	LG3: TA125‐TA34 LG4: TA2‐TA72 LG6: GA26‐TA80	Taleei et al. (2010)
ILC3279	C 214	LG4: AB‐Q‐SR‐4‐1, AB‐Q‐SR‐4‐2 LG5: AB‐Q‐APR‐5B LG6: AB‐Q‐APR‐6‐1, AB‐Q‐APR‐6‐2	AB‐Q‐SR‐4‐1: STMS11‐TA130 AB‐Q‐SR‐4‐2: H4G11‐CaM2049 AB‐Q‐APR‐5B: CaM0038‐CaM0805 AB‐Q‐APR‐6‐1: H1I16‐TA106 AB‐Q‐APR‐6‐2: TA106‐CaM0244	Sabbavarapu et al. (2013)
ICC3996	Lasseter	LG4.1: ab_QTL1 LG4.2: ab_QTL2	ab_QTL1: TA146‐SNP_40000185 ab_QTL2: SNP_40000840‐ SNP_40001505	Stephens et al. (2014)
S95362	Howzat	LG4: ab_QTL1	ab_QTL1: TA146–TA72	
ILC72	Cr5‐10 (a selection from ICCW45 = PI599072)	LG2: QTL_AR3_	QTL_AR3_: GA16‐TA194	Madrid et al. (2014)
CDC Frontier	ICCV96029	LG1: qtlAb‐1.1 LG2: qtlAb‐2.1 LG3: qtlAb‐3.1 LG4: qtlAb‐4.1 LG6: qtlAb‐6.1 LG7: qtlAb‐7.1 LG8: qtlAb‐8.1, qtlAb‐8.2, qtlAb‐8.3	qtlAb‐1.1: CAV1SC21.1P1495114 qtlAb‐2.1: scaffold905p1129574 qtlAb‐3.1: CAV1SC548.1P43520 qtlAb‐4.1: scaffold405p948196 qtlAb‐6.1: CAV1sc445.1p92883 qtlAb‐7.1: CAV1SC102.1P548827 qtlAb‐8.1: CAV1SC679.1P39451 qtlAb‐8.2: scaffold1567p981540 qtlAb‐8.3: scaffold21p63604	Daba et al. (2016)
FLIP84‐92C	PI359075 and PI599072	LG4: qABR4.1, qABR4.2, qABR4.3	qABR4.1: CaNIP8	Kumar et al. (2018)
CDC Frontier	ICCV96029	LG1: qAB1.1, qAB1.2, qAB1.3, qAB1.4 LG4: qAB4.1, qAB4.2, qAB4.3, qAB4.4, qAB4.5 LG6: qAB6.1, qAB6.2	KASP marker	Deokar, Sagi, Daba, et al. (2019)
Amit	ICCV96029	LG2: qAB2.1, Ca2v2.6p18233152_G/A, Ca2v2.6p18250143_T/A, LG4: qAB4.1, qAB4.2, qAB4.3, qAB4.4 LG7: qAB7.1	qAB2.1: Ca2v2.6p18233152_G/A, Ca2v2.6p18250143_T/A, Ca2v2.6p18266481_A/C, qAB4.1: Ca4v2.6p26669292_T/G, qAB4.2: Ca4v2.6p28791114_G/C qAB4.5: Ca4v2.6p43806808_A/G	
Amit	ICCV96029	LG2: qAB2.1, qAB2.2, qAB2.3 LG3: qAB3.1 LG4: qAB4.1, qAB4.2 LG5: qAB5.1 LG6: qAB6.1	qAB2.1: Ca2‐ABA‐RCav1sc520.1p50440 qAB2.2: Cav1sc246.1p121732‐Cav1sc689.1p195825 qAB2.3: Ca2‐GDSL2‐ Ca2‐PEI qAB3.1: SCA3_15444471‐ SCA3_21346384,	Deokar, Sagi and Tar'an (2019)
GPF2	ILWC292 (*C*. *reticulatum*)	LG4: qab‐4.1, qab‐4.2LG7: qab‐7.1	qab‐4.1: CNC_021163.1.32280291, CNC_021163.1.37933917 qab‐4.2: CNC_021163.1.23799836 CNC_021163.1.24184658 qab‐7.1: CNC_021166.1.34330294 CNC_021166.1.34330283	Kushwah et al. (2021)
04067–81–2‐1‐1 (*C*. *echinospermum*)	Sonali	LG4	Ca_Ce_18445 [Ca_Ce_18577 & Ca_Ce_18594] Ca_Ce_18656	Sudheesh et al. (2021)

In the last decade, markers associated with AB resistance have been developed but their use remained low as inferred from publications regarding new varieties of chickpea. The QTLs for AB disease resistance and the double podding trait were successfully introgressed by marker‐assisted backcrossing (Taran et al., 2013). In the elite C214 cultivar, the QTL regions for AB disease and Fusarium wilt race 1 resistance were introgressed by marker‐assisted backcrossing (Varshney, Mohan, et al., 2014). Castro et al. (2015) concluded that marker‐assisted backcrossing is superior to conventional backcrossing as it helps in retaining the elite cultivar's genetic background, which is useful in introgression of multiple useful alleles from different genotypes, and breeding is less influenced by environmental factors, particularly in AB disease‐like fungal diseases. Three AB disease resistance QTLs were identified in a narrowed genomic region by the mQTL‐seq method and the codominant markers (CaNIP8 and CaETR‐1) were developed for the major QTL, qABR4.1. These markers should facilitate AB resistance breeding (Kumar et al., 2018; Madrid et al., 2013). We hope that in the near future chickpea AB disease resistance will be managed by faster breeding of resistant cultivars through advanced technologies.

## 8 CONCLUSION AND FUTURE RESEARCH DIRECTIONS

AB disease is a serious threat to global chickpea production. Constant surveillance of AB disease emergence is required in countries from where seeds are being heavily imported. In order to understand the evolution of aggressive isolates under the selection pressure of resistant cultivars and novel fungicides, analysis of pathogen population structures, host–pathogen relationships, and diversity by next‐generation sequencing is essential. It is expected that new technologies will be used to elucidate the genetic makeup of pathogens and compare their pathogenicity. One of the most important factors contributing to rapid fungal evolution and adaptation may lie in their ability to reproduce sexually. Further field samples need to be analysed to test for the presence of both mating types and extensive efforts should be made to prevent the introduction of a second mating type in areas where one mating type is lacking. More options should be added to achieve integrated disease management. Sharing of isolates' phenotyping and genotyping data should be encouraged so that comparative analysis among isolates of various countries can be performed. This will result in a better understanding of *A*. *rabiei* evolution under AB‐resistant/tolerant host selection pressure, diverse environmental conditions, and fungicide treatment. Future research of AB disease‐causing fungal pathogens should be concentrated on the following areas:

- High‐throughput phenotypic and genotypic analyses should be conducted in order to better understand the genes that are evolving with the emergence of aggressive isolates.
- A uniform pathogenicity classification system should be established, at least for comparison of isolates' aggressiveness. The best‐suited germplasm screening methods as per the environmental conditions of each region should be adopted.
- Genes that make *Ascochyta* spp. adapted to a particular legume host can now be analysed in a better way with the availability of new rapid and economical genotyping technologies along with in vitro mating protocols.
- Transgenic chickpea plants could be used to maintain a repertoire of validated potential targets of *A*. *rabiei* for host‐induced gene silencing. The use of the clustered regularly interspaced short palindromic repeats (CRISPR) system in *A*. *rabiei* should be encouraged for rapid functional analysis of genes.

## Data Availability

Data sharing is not applicable to this article as no new data were created or analysed in this study.
